# Traumatic Pneumothorax Secondary to Acupuncture Needling

**DOI:** 10.7759/cureus.3194

**Published:** 2018-08-23

**Authors:** Ching-Hui Sia, Aloysius Sheng-Ting Leow, Benjamin Sieu-Hon Leong

**Affiliations:** 1 Department of Cardiology, National University Health System, Singapore, Singapore, SGP; 2 Yong Loo Lin School of Medicine, Singapore, SGP; 3 Department of Emergency Medicine, National University Health System, Singapore, SGP

**Keywords:** acupuncture, chest pain, needling, pneumothorax

## Abstract

Acupuncture is a common form of therapy involving insertion of fine needles to alleviate nausea and various forms of pain. We describe a case of pneumothorax secondary to acupuncture.

A 50-year-old woman presented to the emergency department with right-sided pleuritic chest pain. This was following a history of acupuncture and cupping treatment an hour earlier at a traditional practitioner for long-standing neck pain. On physical examination, the respiratory rate was 22 breaths per minute and her oxygen saturation was 100% on room air. Breath sounds were decreased on the right hemithorax with hyper resonance to percussion. Inspection of her back revealed multiple needling and cupping marks. A chest radiograph revealed a right-sided pneumothorax with an apex-cupola distance of 3.6 cm. She was put on high flow oxygen and a chest tube was inserted into the right chest wall. The patient was admitted. She had radiographic resolution of the pneumothorax four days later and was discharged uneventfully. Follow-up one week later in the clinic showed no radiographic recurrence of the pneumothorax.

## Introduction

Acupuncture is a form of therapy involving insertion of fine needles into specific sites on the body within the subcutaneous layer [1]. While commonly used to alleviate nausea [2] and various forms of pain [3,4], serious adverse events such as pneumothorax, septicemia and spinal cord injury should not be overlooked [5]. The following case report describes a case of pneumothorax secondary to acupuncture.

## Case presentation

A 50-year-old Chinese lady presented to our emergency department with right-sided chest pain. She had a history of De Quervain’s tenosynovitis of the left hand post-release seven years earlier and was a non-smoker.

She complained of right-sided chest pain on deep inspiration. She had just sought treatment at a traditional medicine practitioner for a session of acupuncture and cupping (‘ba guan’) to the back to treat her symptoms of long-standing neck pain and intermittent numbness and tingling of both hands. This pain occurred while she was sitting down after receiving treatment. She denies any trauma to her chest. She did have some difficulty taking in breaths due to the chest pain. There was no fever, cough, runny nose or sore throat.

**Figure 1:**
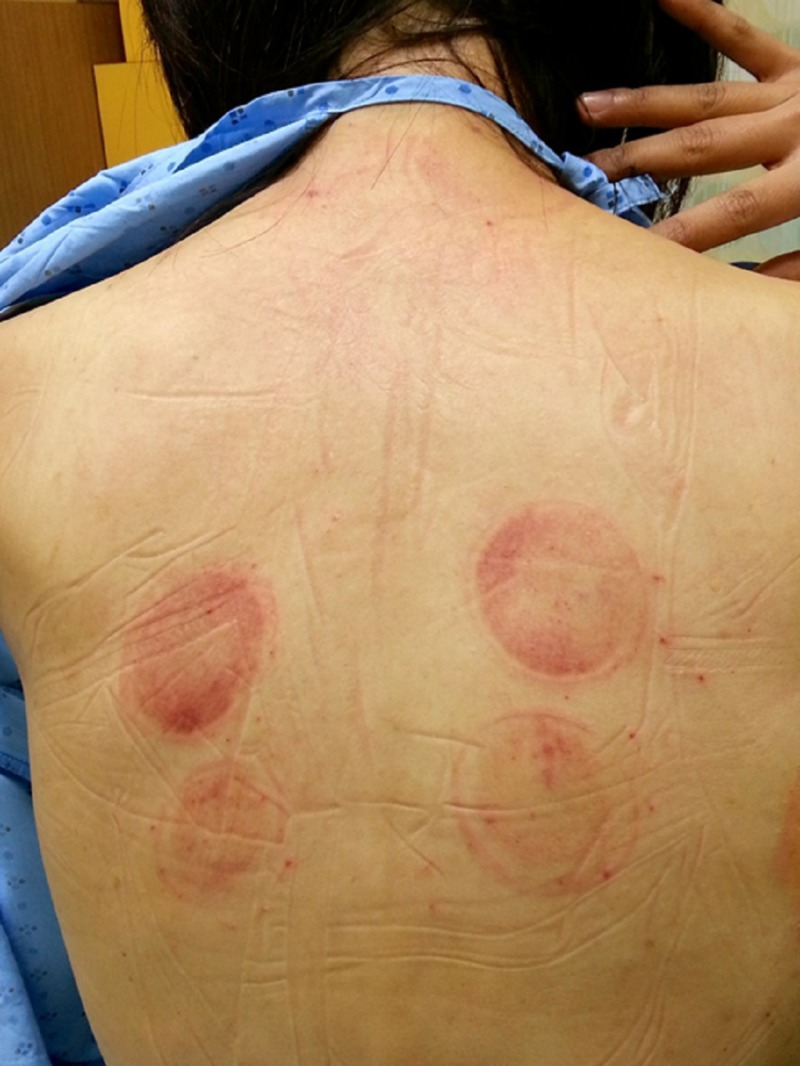
Back of the patient on initial examination.

Physical examination revealed that she was afebrile, had a blood pressure of 99/63 mmHg, heart rate of 96 beats per minute, respiratory rate of 22 per minute and an oxygen saturation of 100% on room air. She was alert, comfortable and conversant in full sentences. Her heart sounds were dual, with no clicks, rubs or murmurs. Breath sounds were slightly decreased on the right but otherwise no crepitations were heard. Inspection of her back is as shown in Figure 1, with multiple cupping and needling marks. Her abdomen was soft and non-tender. She had supple calves and no pedal edema on examination.

**Figure 2:**
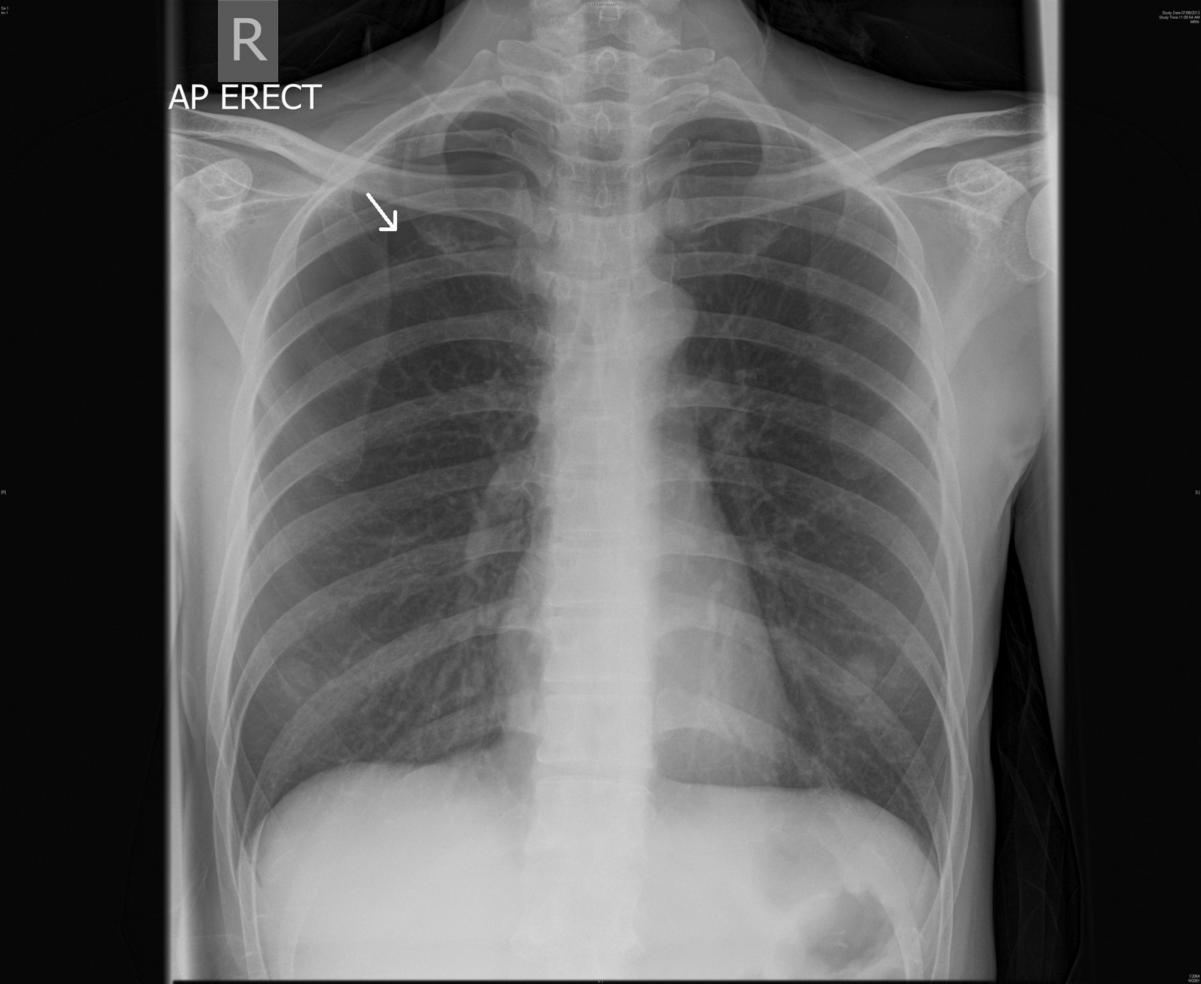
Right apical pneumothorax with apex-cupola distance of 3.6 cm.

Initial investigations revealed a normal complete blood count and metabolic panel. Electrocardiogram showed normal sinus rhythm with no acute ST segment or T wave changes. The chest radiograph on admission is as shown in Figure 2, with a right-sided pneumothorax with an apical-cupola distance of 3.6 cm.

**Figure 3:**
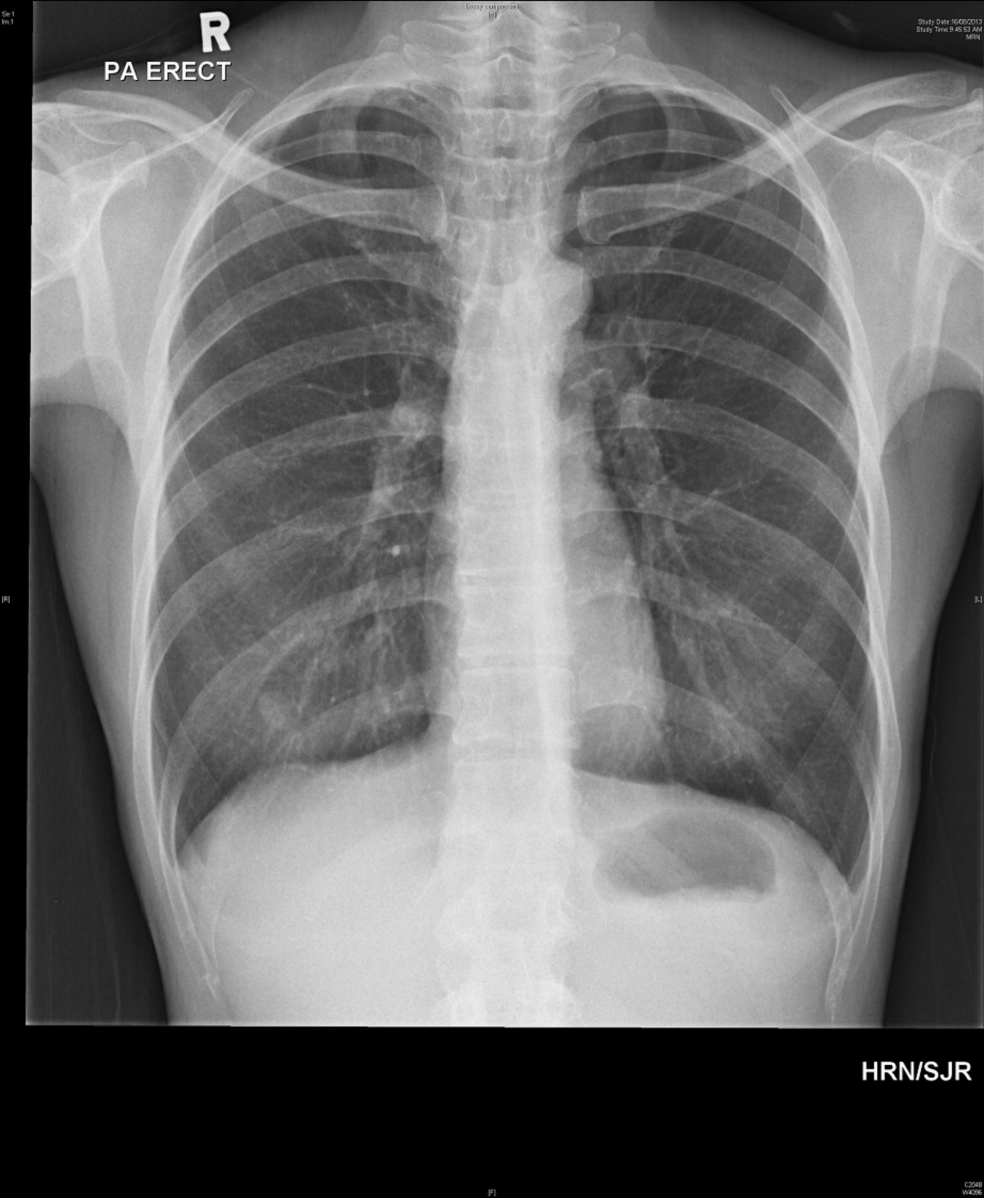
Resolution of pneumothorax on follow-up.

Subsequently, the patient was put on a non-rebreather mask with a FiO2 of 100% and a 12 French chest tube was inserted via the Seldinger’s technique into the right chest wall in the emergency department. The patient was admitted to the Respiratory service with radiographic resolution of the pneumothorax within four days and was discharged when the apex-cupola distance of the pneumothorax was 4 mm. A repeat chest radiograph a week later in the follow-up clinic showed complete resolution of the pneumothorax (Figure 3).

## Discussion

We report a rare case of a traumatic pneumothorax secondary to acupuncture needling. The exact incidence of this condition is unknown, but a systematic review has estimated it to occur in twice in nearly a quarter of a million treatments [6]. Based on our knowledge, this is the first published image of a patient’s back together with a corresponding chest radiograph.

Acupuncture is widely considered to be a safe alternative to Western medicine and there has been increasing interest by patients in seeking this form of treatment for their ailments. Common uses (not necessarily evidence-based) include management for pain, addiction management, menopausal symptom control, for cancer and post-operative conditions [2-5].

However, considerable serious adverse events exist which the medical practitioner should know about. Common events include fainting, nausea and vomiting, pain, diarrhea, local skin bruising or bleeding, psychiatric disturbances, headaches, sweating, dizziness, aggravation of symptoms or needle breakage. More serious complications reported include pneumothorax (with or without tension), spinal cord injury, cardiac tamponade, staphylococcal septicemia, hepatitis B/C/HIV infection, convulsions and even death [5]. Su et al. presented a case of bilateral tension pneumothorax in an unfortunate lady at their institution [7].

In cases of suspected pneumothorax associated with acupuncture, we suggest performing a thorough physical examination including inspection of the back to look for any needling marks. This is often overlooked in a busy clinical environment such as the emergency department or when the patient is clothed. A review by the World Health Organization on complications of acupuncture showed that acupuncture-associated pneumothoraces had puncture sites mainly in the shoulder and scapular regions (64%) and in the chest (24%) [8]. This corresponded with our case. As immediate radiography of the chest may not reveal the presence of the pneumothorax, a repeat chest radiograph after 24 hours is recommended if suspicion is high [9]. On the other hand, various studies have reported higher sensitivity of point-of-care ultrasound than chest radiograph in identifying pneumothorax, which underlies its increasing use these days and would be a valuable modality when evaluating patients with suspected pneumothorax [10,11].

## Conclusions

Recent history of treatment by a traditional medicine practitioner should raise one’s suspicion for acupuncture-associated pneumothorax and requires careful history and physical examination to correlate onset of symptoms with the procedure.
